# Oral microbiome: possible harbinger for children’s health

**DOI:** 10.1038/s41368-020-0082-x

**Published:** 2020-04-30

**Authors:** Jin Xiao, Kevin A. Fiscella, Steven R. Gill

**Affiliations:** 1 Eastman Institute for Oral Health, University of Rochester Medical Center, Rochester, NY USA; 2 Department of Family Medicine, University of Rochester Medical Center, Rochester, NY USA; 3 Department of Microbiology and Immunology, University of Rochester Medical Center, Rochester, NY USA; 4 Genomics Research Center, University of Rochester Medical Center, Rochester, NY USA

**Keywords:** Microbiome, Paediatric research

## Abstract

The human microbiome functions as an intricate and coordinated microbial network, residing throughout the mucosal surfaces of the skin, oral cavity, gastrointestinal tract, respiratory tract, and reproductive system. The oral microbiome encompasses a highly diverse microbiota, consisting of over 700 microorganisms, including bacteria, fungi, and viruses. As our understanding of the relationship between the oral microbiome and human health has evolved, we have identified a diverse array of oral and systemic diseases associated with this microbial community, including but not limited to caries, periodontal diseases, oral cancer, colorectal cancer, pancreatic cancer, and inflammatory bowel syndrome. The potential predictive relationship between the oral microbiota and these human diseases suggests that the oral cavity is an ideal site for disease diagnosis and development of rapid point-of-care tests. The oral cavity is easily accessible with a non-invasive collection of biological samples. We can envision a future where early life salivary diagnostic tools will be used to predict and prevent future disease via analyzing and shaping the infant’s oral microbiome. In this review, we present evidence for the establishment of the oral microbiome during early childhood, the capability of using childhood oral microbiome to predict future oral and systemic diseases, and the limitations of the current evidence.

## Introduction

Human life is dependent on a diverse community of symbiotic microbiota that has co-evolved with their human host. The interactions between microbiota and host modulate crucial aspects of the host’s normal physiology, metabolism, immunity, and neurologic function.^1^ In the adult population, the host-microbiome at various body sites has reported associations with systemic diseases. For instance, dysbiosis in the gut microbiome could induce an ecological shift of the microbial community, from a physiological to a pathological composition, lead to dysregulated production of harmful microbial-derived products or metabolites, and contribute to or become a risk marker for a diverse range of local and systemic diseases. The reported diseases that have a gut microbial association include but not limited to inflammatory bowel diseases.^2,3^ celiac disease (CD),^4,5^ depression.^6^and Alzheimer’s disease.^7^ Intriguingly, an intricate linkage between the dysregulated gut microbiome and osteoarthritis was recently revealed. The loss of beneficial *Bifidobacteria* while an increased abundance of key proinflammatory species in the gut microbiome is found to be associated with obesity; this microbial imbalance event is further linked with a downstream of systematic inflammation that could accelerate knee osteoarthritis.^8^ Associations between oral microorganisms and cancers (oral and esophagus,^9–12^ pancreatic,^13^ and colorectal^14^) have also been suggested recently. Moreover, periodontal microorganisms, i.e., *Porphyromonas gingivalis, Aggregatibacter actinomycetemcomitans*, *Provetella intermedia*, have been found in human atheromatous plaques at various sites, with implied association with vascular disease.^15^ Furthermore, a dysbiosis in the vaginal microbiome is associated with a variety of adverse outcomes, including miscarriage, invasive maternal and neonatal infections, and preterm birth delivery.^16–20^

Through the first years of life, the newborn infant microbiota is highly dynamic and undergoes rapid changes in composition, towards a stable adult-like structure that harbors distinct microbial communities of unique composition and functions at specific body sites.^21–28^ Colonization of oral mucosal surfaces begins at birth with the introduction of bacteria and fungi through multiple paths, including maternal transmission during childbirth, parental exposures, diet and horizontal transmission from caregivers and peers.^29–32^ The oral microbial community continues to develop with the eruption of primary teeth in early infancy and establishment of permanent dentition in children, evolves into a complex and diverse microbiome.^33^ A complex interplay between establishment and development of the neonate’s immunity and early microbial acquisition occurs.^34,35^ These early life interactions between the microbiome and human host are responsible for features of postnatal innate and acquired immune functions and physiological development that influence future health.^28,36–38^

The oral cavity serves an initial entry point for colonization of the oral and gut microbiota^39,40^ and therefore is an easily accessed body site for assessment of the microbial community, and biologic markers used to diagnose, predict, and monitor both oral and systemic diseases.^41^ Similar to reported associations between microbiome and adults’ health, recent data suggest that disruptions in early oral colonization and establishment of a healthy oral microbiome may influence the progression of both oral and systemic conditions in children. Despite that more longitudinal studies are critically needed to provide substantial evidence on causal relationship between the oral microbiome and oral/overall health, health conditions that potentially have an oral microbial involvement and harbor oral microbial signatures include but not limited to children’s tooth decay,^42–48^ infant weight gain,^40^ pediatric appendicitis,^49^ and pediatric inflammatory bowel disease.^50^

To the vulnerable populations, infants and young children, oral sample collection in forms of saliva and mucosal swabs is noninvasive and thus presents an optimal diagnostic medium that holds great promise for use as diagnostic tools. Moreover, researchers are searching for the potential utility of the microbiome with a particular focus on manipulating microbials.^51^ Microbial intervention of a single bacterial strain was demonstrated to be effective in altering disease risk in the low-complexity microbiome of the newborn gut.^52^ Although, to date, no studies have addressed the impact of manipulation of the oral microbiome on systemic disease, as the microbiome research moves on a fast track, we can envision a future where early life salivary diagnostic tools could be used to understand better and shape our oral and systemic health. Therefore, this review attempts to gather evidence and elucidate the establishment of the oral microbiome during early childhood, the factors associated with oral microbial profiling, and the capability and challenges of using childhood oral microbiome to predict future oral and systemic diseases.

## Oral microbial community establishment in early childhood

Several terms are used to define different stages of early childhood. Medically, the newborn or neonate is defined as an infant in the first 28 days after birth. The term “infant” typically refers to young children under 1 year of age, or some definitions expand this period to up to 2 years of age. When a child learns to walk from age 1 to 4, the term “toddler” may be used instead. The period of birth to preschool age (5–6 years old) is considered as early childhood.

Oral microbial colonization is traditionally considered to take place after birth; however, recent studies have brought our attention to the commence of the human microbiome before birth. Studies reported the presence of microorganisms in amniotic fluid in up to 70% of the pregnant women, and particularly the presence of several oral microorganisms, such as *Streptococcus*, *Fusobacterium*, *Neisseria*, *Prevotella*, and *Porphyromonas*, in the human placenta.^53,54^ To assess the possible origin of the placental microbiome, Gomez-Arango et al.^55^ examined the gut, oral, and the placental microbiome from pregnant women using 16S rRNA sequencing. Three shared genera, including *Prevotella, Streptococcus*, and *Veillonella*, were found in all gut, oral, and placenta samples.^55^ Surprisingly, the placenta microbiome did not harbor unique core genera when the microorganisms residing in the gut and oral cavity were compared.^55^ Moreover, although the placental colonization may have multiple sources (oral and gut), the placental microbiome resembles more like the pregnant oral microbiome.^55^

**Fig. 1:**
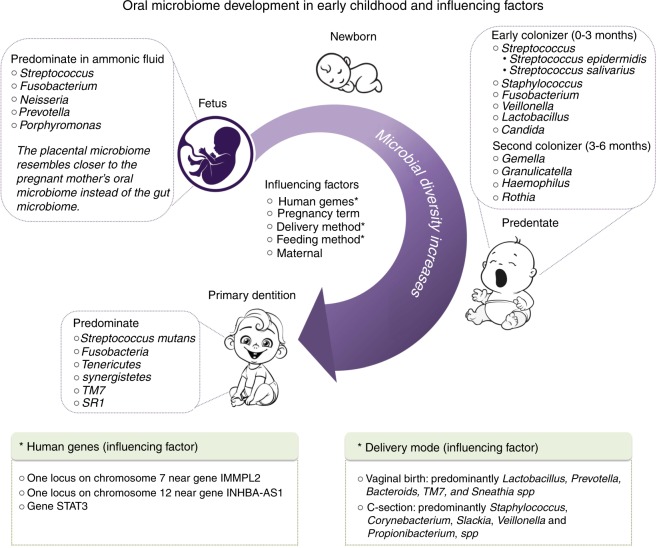
Oral microbiome development in early childhood and its influencing factors

During and after birth, the newborn exposes to a wide variety of microorganisms, e.g., bacteria, fungi, parasites, and virus, under the influence of contact routes and an infant’s immune tolerance.^56^ Remarkably, the oral microbial colonization follows a temporal and spatial sequential, and only a subgroup of microorganisms become permanent residents of the oral cavity,^57^ illustrated in Fig. 1. The set of earlier colonizers seems to condition the subsequent colonization, which leads to more complex and stable ecosystems in adulthood.^58^

### Oral bacterial community

Immediately after birth, with contact with the outside world through breathing, feeding, and the contact with care providers, the colonization and establishment of microbial pioneers in the oral cavity take the stage. In a cross-sectional designed study, Mason et al.^29^ analyzed the oral mucosal swab samples from 47 infants at the prdentate stage and identified 178 species-level operational taxonomic units (s-OTUs) that belong to 50 genera, with (65 ± 18) s-OTUs in each infant. The majority of these identified species were Gram-positive facultative, followed by Gram-negative anaerobes.^.29^ Mason et al.^29^ further identified a core of oral microbiome for predentate infants, including genera *Streptococcus*, *Gemella*, *Granulicatella*, and *Veillonella*. Interestingly, these core species only accounted for 45% (23%–61%) of the total oral microbiota of each infant.^29^ Out of the 178 identified species, only 33 were shared by ≥75% of infants. The mean number of shared core species by each infant was 27 ± 5.^29^

As an infant grows, oral bacterial diversity and richness continue to increase through time. Through sequencing of 16S rRNA gene V3–V4 hypervariable regions, Dzidic et al.^59^ examined the temporal evolution and maturation of the oral microbial ecosystem in early infancy and childhood using a longitudinally collected oral sample set from 90 children. These children were followed from birth to 7 years of age. Before tooth eruption, the mucosal surfaces serve as primary sites for bacterial colonization. The most frequently detected early colonizers in the oral cavity are *Streptococcus* (*Streptococcus epidermidis* and *Streptococcus salivarius*), *Staphylococcus* spp., and *Fusobacterium.*^.29,59^ The reasons for the high abundance of *Streptococcus* in the early oral cavity lie in: (a) *Streptococcus* spp. are capable of adhering to epithelial cells and (b) *Streptococcus* spp. are one of the dominant bacterial groups found in human breastmilk; the oral settlement of *Streptococcus* spp is initiated by direct transmission via physical contact through bread feeding, facilitated by an appropriate nutrient supply in breast milk that favors the growth of *Streptococcus* spp, and further reinforced by repeated breast milk feeding.^60^ Among *Streptococcus* species, *Streptococcus salivarius* is the most often found species in the oral cavity of the newborn^61,62^, its abundance reaches the highest at 3 months of age, accounting for 10%–15% of the total *Streptococcal* species. The abundance of *S. salivarius* decreases steadily its peak at 3 months, likely opposing to teeth eruption.^30^ In addition to the early colonizers, bacterial genera *Gemella*, *Granulicatella*, *Haemophilus*, and *Rothia* present at 3–6 months of age with more than 1% of abundance; and their abundance further increases with time.^59^

Remarkably, the oral ecosystem reforms with the eruption of the first tooth. The salivary microbial community of the primary dentition reveals a significant greater alpha diversity and equitability when compared to that of the predentate infants.^29^ A lower level of Gram-positive facultative and a higher level of Gram-negative facultative are seen when transitioning from the predentate to primary dentition.^29^ For instance, in the first several months of an infant’s life, before tooth eruption, *Escherichia coli*, *Pseudomonas*, *Staphylococcus*, along with the lactic acid-producing bacteria such as *Lactobacillus gasseri*, *Lactobacillus crispatus*, and *Streptococcus* spp are highly prevalent.^61,63,64^ Along with the tooth eruption providing new binding sites, new ecological events take place in the oral environment. *Streptococcus mutans*, for example, accelerates its colonization at this stage, due to the emergency of their preferable adhesion surface, teeth. As time goes by, *Fusobacteria*, *Synergistetes*, *Tenericutes*, *TM7*, and *SR1* dominate the oral microbial community by the end of first year of life.^65^ Post tooth eruption, although most oral microorganisms colonize all oral cavity sites, including mucosa, tongue, and teeth; their proportion may differ depending on the colonization sites. A higher microbial load is found on the teeth and tongue comparing to the oral mucosa and saliva. In parallel to developing specific microbial niche at different oral sites, the sral bacterial diversity and richness continue to increase through time. At the end of primary dentition, 7 years of age, nearly 550 OTUs were identified in the oral cavity; the Shannon diversity index reaches approximately 2.4 (ref. ^59^).

After tooth eruption, the plaque microbiota forms a new, distinct oral community. As noted in our study, while comparing the oral microbial community between preschool children with and without caries using 16s sequencing, the microbial composition differed notably between saliva and plaque samples, regardless of caries status.^48^ At the genus level, the two most abundant genera in children’s saliva were *Streptococcus* and *Veillonella*, whereas, in plaque, the most abundant genera were *Veillonella*, *Streptococcus, Actinomyces, Selenomonas*, and *Leptotrichia.*^48^ At the species level, three dominant taxa represented 60% of the relative abundance in the salivary community: *Streptococcus ET_G_4d04, Veillonella atypica_dispar_parvula*, and *Streptococcus vestibularis_salivarius.*^48^ In contrast, the five most abundant species, representing 30%–50% of the bacterial plaque community, were *Veillonella atypica_dispar_parvula, S. mutans, S. ET_G_4d04, Streptococcus oral_taxon_B66*, and *Streptococcus gordonii.*^4^

### Oral fungal community

In addition to bacterial community, the oral cavity of newborns is also known to be colonized by fungal community, specifically *Candida*, starting on the first day of life; during the first year, the rate of oral colonization by *Candida* may vary, depending on the study population and detection methods, the detection rate range between 40% and 82%.^66–68^ For an extended period, *Candida* was the only fungus recognized as part of the normal oral microbial population, despite its opportunistic character.^69^ In 2010, a metagenomic study identified 74 genera of cultivable fungi and 11 uncultivable fungi in the oral cavity of healthy adults.^70^ Although *Candida* was the most frequent genus isolated in 75% of the subjects, other fungi groups presented a relevant prevalence, such as *Cladosporium* (65%), *Aureobasidium* (50%), *Saccharomycetales* (50%), *Aspergillus* (35%), *Fusarium* (30%), and *Cryptococcus* (20%).^70^

Most recently, the skin, oral, and anal mycobiomes were analyzed by Ward et al.^32^ among an infant cohort from birth through 30 days of life, using amplicon sequencing of internal transcribed spacer 2 (ITS2). Ward et al.^32^ noted that between colonization sites, the alpha diversity of the oral mycobiome was significantly lower than that of skin and anal mycobiomes. Within individuals, the infant oral mycobiomes exhibited high intra-individual variability for beta diversities over time; however, when measured by weighted UniFrac distances, no distinct clusters were identified.^32^ Mycobial community differs across the body sites. On infant skin, the most abundant and prevalent mycobial taxa were *C. tropicalis*, *C. parapsilosis*, *S. cerevisiae*, *C. albicans*, and *C. orthopsilosis*; in infant oral cavity, the abundant and prevalent taxa were *C. parapsilosis*, *C. tropicalis*, *S. cerevisiae*, *C. orthopsilosis*, *C. albicans*, and *Cladosporium velox*; among the infant anal mycobiome, the most abundant taxa were *C. parapsilosis*, *C. tropicalis*, *C. albicans*, *S. cerevisiae*, *C. orthopsilosis*, and *Cryptococcus pseudolongus*.

Nevertheless, since there have been few reported longitudinal studies of mycobial community in early infancy, the role of this oral “mycobiome” and their identification in the oral cavity of children remains underexploited.

### Oral viral community

Viruses have also been found in the oral cavity, with their presence primarily viewed as a pathological nature. In the past decade, there have been only a handful of publications reporting the oral virome, which is in contrast to the oral microbiome, an area that demands critical attention. The oral cavity harbors 300–2 000 viral genotypes in any individual.^71^ The most common viruses isolated from the oral cavity include rotavirus, norovirus, HIV, hepatitis C virus, herpes simplex viruses 1 (HSV1) and HSV2, Epstein–Barr virus and influenza viruses.^72^ In addition to these commonly reported virus, some less abundant viruses, e.g. eukaryotic DNA viruses including herpesvirus HPV7 and Anelloviruses, as well as some RNA viruses, are also permanent constituents of human mouth.^72,73^

Several viral agents can infect oral mucosal cells; however, only a few cause clinical manifestations. In children, the severity of symptoms is related to the age at which the infection was acquired. Newborns could be infected by HSV1 and HSV2, which can cause herpetic gingivostomatitis, orofacial herpes, and aphthous stomatitis.^74^ Neonatal HSV infection is uncommon, with the reported incidence rates as 1.6–33 per 100 000 live births.^75^ HSV infection in a newborn can be very severe and even cause death due to the compromised and underdeveloped infant immune systems.^76^ Other viruses that could colonize in the oral cavity during early childhood are the Coxsackie A virus, which causes herpangina and hand, foot, and mouth disease; the Morbillivirus that causes measles; the Rubulavirus that causes mumps; and the human papillomavirus that causes oral papilloma (warts).^77^

## Factors shaping children’s oral microbiome

For healthy adults, an individual’s oral microbiome is associated with many factors, including the time of the sample collection, age, gender, diet, extreme environment, etc.^78^. Similarly, the host and environmental factors also influence the assembly of the oral microbiome in early childhood. The most studied factors include genetics, terms of labor, delivery mode, antibiotics use during birth and early infancy, feeding method, and maternal oral microbiome characteristics. These influencing factors contribute to shaping both bacterial and fungal communities.^78,79^ The summary in Fig. 1 highlights the factors that shape children’s oral microbiome.

### Genetic determinants

Even though few studies have identified genes that contribute to microorganism infections,^80^ the influence of human genes on the colonization and establishment of the oral microbiome remains under-investigated. Demmitt et al.^81^ examined the contributions of host genetics and environmental factors to salivary microbiome composition using an unbiased genome-wide association study (GWAS) analysis in 752 twin pairs from the Colorado Twin Registry. A method that used KGG4 that sums single-nucleotide polymorphisms (SNP) significance across coding genes were used. Several loci reached genome-wide significance after correcting for multiple testing, including one locus on chromosome 7 near the gene IMMPL2 and one locus on chromosome 12 near the non-coding RNA gene INHBA-AS1 (ref. ^81^).

Exploring the effects of host genotype on the oral microbiome composition and caries phenotypes also gained attention. While several heritable oral taxa were identified, the scientific finding has not reached agreement on whether the abundance of traditionally considered cariogenic bacteria hold inheritable traits. Gomez et al.^82^ profiled the supragingival plaque microbiome from 485 dizygotic and monozygotic twins, with a mean age of (7.8 ± 1.4) years old. This research group revealed that the similarity of the oral microbiota increased with a shared host genotype, regardless of caries status. Interestingly, the most heritable oral bacteria, such as *P. pallens*, were not associated with caries status but associated with age and sugar consumption. *P. pallens* decreased in abundance when children are older and sugar consumption frequency increase. However, the results from Gomez et al. stating that some the abundance of certain cariogenic bacteria are lack of heritable traits are in contrary to some other studies. Previous twin studies used culture- and array-based approaches to obtain microbial information and examined the heritability of oral microbes. These studies resulted indicated that the abundance of the cariogenic *S. mutans* and specific pathogenic attributes such as acid production in dental plaque and saliva have a close genetic correlation.^83,84^ Furthermore, the abundances of *Prevotella*, *Pasteurellaceae*, and *Leptotrichia* have been previously found to be associated with SNPs in host genes that code for ATP-binding cassettes, protein synthesis, cell division, and tumor suppression.^83^

Human genetics also influence individual’s susceptibility to fungal infection. Abusleme et al.^85^ demonstrated that individuals with defects in STAT3 are prone to the dysbiosis of oral fungal and bacterial community, and susceptible to recurrent oral fungal infections. STAT3 is a protein coding gene, playing a critical role as transcription factors in the downstream of cytokine signaling from interleukin-6 (IL-6), IL-21, IL-10, and IL-23—a group of human immune mediators. Patients with autosomal-dominant hyper-IgE syndrome (AD-HIES) harbor loss-of-function (LOF) mutations in the *STAT3* gene.^86,87^ In turn, a severe dysbiosis of fungal community was found in AD-HIES patients’ oral mucosa, with a dominance of *C. albicans* and minimal representation of health-associated fungi.^85^ The STAT3 defect in participants with AD-HIES was also associated with distinctive bacterial communities, particularly with a reduced microbial diversity and an enrichment of *Streptococcus oralis* and *S*. *mutans*. Moreover, increased abundance of cariogenic bacteria—*S*. *mutans*—was consistent with the increased risk for dental caries among patients AD-HIES.^85^

### Preterm and full-term pregnancy

In medical context, preterm refers to the condition that infants born alive before 37 weeks of pregnancy.^88^ Sub-categories of preterm birth, based on gestational age, include extremely preterm (<28 weeks), very preterm (28–32 weeks), and moderate to late preterm (32–37 weeks).^88^ Globally, approximate 15 million babies are born preterm yearly.^89^

Although a small number of studies have compared the oral microbiota among infants with different pregnancy terms, difference in the oral microbiota diversities between the full-term and preterm infants was not discovered. For instance, in a cross-sectional designed study, Younge et al.^90^ characterized the oral and skin microbiota of 89 preterm (23–36 weeks) infants in intensive care unit with an average age of 42 days (1–252), and 40 full-term infants with an average age of 1 day (0–122) during their birth hospitalization. Among the preterm infants, 48% were white, 50% were Black or African American, and 3% were unknown races; among the full-term infants, 51% were white, 31% were Black or African American, and 15% were unknown races.^90^ The samples collected from the oral cavity and various skins sites included the forehead, posterior auricular scalp, periumbilical region, inguinal folds, and upper thighs.^90^ Among the oral microbiota of full-term and preterm infants, no significant difference were found in alpha diversity measured by the Shannon diversity index.^90^ The beta-diversity measured by principal coordinate analysis differed significantly between the oral and stool samples (*P* < 0.000 1), and between the skin of the upper body and oral samples (*P* = 0.004 9).^90^ In contrast to the oral community, the skin bacterial community differed significantly by gestational and postnatal age, and body sites.^90^ Another small-scale study analyzed the oral microbiota of seven preterm infants (25–27 weeks gestational age) at three timepoints in the first five days after birth. This study revealed a transition of the predominate species in the infants’ oral cavity, from a predominance of *Mycoplasmataceae* and *Moraxellaceae* in infants’ first 36 h of life to a predominance of *Staphylococcaceae* and *Planococcaceae* by day 5 (ref. 91).

Concerning enough, when a comparison was made between the full-term and preterm infants with extremely low birth weight (LBW), who was less than 100 0 g in the first 6 weeks of life, preterm with extremely LBW infants were prone to be colonized with pathogenic microorganisms in the oral cavity.^92^ Approximately 20% of the preterm and extreme LBW infants by week 1 and over 50% of the preterm/extreme LBW infants by week 6 were colonized with pathogens, e.g., methicillin-resistant *Staphylococcus aureus* (MRSA).^92^

A critical point to be addressed is while the infants born full-term have ongoing interaction with the mother, family, and home environment, which might affect the early oral microbiome development. Unfortunately, preterm or LBW infants are usually separated from their mothers immediately after birth and are cared by medical personnel in the complex environment of the neonatal intensive care unit (NICU) for a period ranging from days to months. Given NICU has its unique environmental microbes, including commensal and pathogenic organisms,^93,94^ the infant’s ongoing and exclusive interaction with medical personnel, in addition to receiving specific medical procedures and medications in NICU, has the potential to impact preterm and LBW infants’ oral microbial colonization.^95^

Moreover, antibiotic use in NICUs remains widely diverse and controversial. Antibiotics are effective in treating pathogenic bacteria-related infections but simultaneously deplete commensal bacteria and promote the growth of opportunistic fungal species. Studies have shown the association between neonatal antibiotics use and decreased levels of *Clostridial* colonization in infants’ gut when administered in a prolonged period.^96^ Intriguingly, a study by Gomez-Arango et al.^97^ indicated that maternal antibiotics use during delivery also impacts the infant oral microbiome development. In this study where the infant oral swabs were sampled in the first three days after delivery, the study results showed that *Proteobacteria* were abundant in infants with maternal intrapartum antibiotics exposure while *Streptococcaceae*, *Gemellaceae*, and *Lactobacillales* were dominant in unexposed neonates.^97^

### Delivery mode

Similar to what occurs to gut microbiota in early infancy, some studies found that the delivery modes—vaginal delivery or cesarean section—also influence the oral microbial community development in infants; however, the term of influence remains unclear. Immediately after birth, the bacterial niche in different habitats of the newborn (oral, nasopharyngeal, skin, and intestines) are very similar to each other.^23^ Nevertheless, as time goes by, bacterial communities colonized on infants born vaginally resemble mothers’ vaginal bacterial communities, predominantly *Lactobacillus*, *Prevotella*, *Bacteroids*, *TM7*, and *Sneathia* spp., whereas the bacterial profile of infants born by cesarean section resemble those present in mothers’ skin, predominantly *Staphylococcus*, *Corynebacterium*, *Slackia*, *Veillonella*, and *Propionibacterium* spp.^23,98,99^

A birth cohort study conducted in Ireland among 84 infants from birth to the first year of life revealed that birth mode did pose an impact on the infant microbiota, but only up to the first week of age.^100^ The influence disappeared beyond the first week after birth.^100^ Within the first week, higher bacterial community diversity measured by Shannon index (*P* = 0.037) and PerMANOVA assay (*P* = 0.047) was detected in the C-section group than those in the vaginally delivered infants.^100^ On the contrast, a Sweden birth cohort of 59 infants did not reveal associations between birth route and young children’s oral microbiome.^101^ The difference in the bacterial community in early infancy that associated with birth route might remain or reduce, or might even have long-term effects on childhood oral microbiome composition, metabolism, and implications to overall health.^102^ However, these projections still need to confirmed by more well-designed longitudinal birth studies.

### Feeding method

Similar to the influence of feeding methods on the gut microbiota,^103^ the oral microbiota of formula-fed and breastfed infants have distinct characteirstics.^104^ The difference in infants’ oral microbiome may arise due to the milk-related bacteria transmission,^105^ effects of various milk components on the attachment of bacteria in the oral cavity,^106^ differed utilization of carbohydrates in breastmilk and formula by bacteria, and/or infant’s boosted innate immunity from breast milk and its effect on early oral microorganisms colonization.^107^

Researchers from Sweden^108^ characterized and compared the oral microbiome in formula-fed and breastfed infants. The oral microbiota pattern of breastfed infants differed markedly from the formula-fed infants, with significantly lower species richness at 4 months of age.^108^ However, notable enough, this difference in oral species richness between breastfed and formula-fed infants disappeared when these infants reached 12 months of age. In contrast to the species richness, the difference of certain microbial community characteristics remained even after the discontinuation of the breastfeeding, which indicates that there might be a long-term effect of breastfeeding on the oral microbiota and this phenomenon deserves further follow-up.

Moreover, this study examined how types of formula could influence the oral microbiota development. An experimental formula that was supplement with bovine milk fat globule membranes was compared to standard formula. Researchers found that although oral species richness did not differ between the experimental and standard formula groups, consumption of the experimental formula was associated with significant differences in taxa abundance, e.g., lower level of *Moraxella catarrhalis* found in the experimental formula group. Furthermore, when infants reached 2 years of age, oral bacterial diversity appears to be higher in children who abandoned breastfeeding before turned 1 year of age.^59^

Regarding *S. mutans*, a bacterial species that is famous for its acidogenicity, aciduricity, and capability of synthesizing extracellular matrix using carbohydrates, although not conclusive, some studies demonstrated that when the presence of *S. mutans* in the oral cavity of 1-year old infants was more prevalent in formula-fed than in breastfed infants.^109^ Previously, in vitro studies revealed that both human and bovine milk may inhibit the metabolism and adhesion of *S. mutans.*^110^ Although the association between breastfeeding and risk of caries is debatable since *S. mutans* is a known culprit for caries, the supporting evidence that formula-fed infants harbor more *S. mutans* at 12 months of age indeed support the beneficial role of breastfeeding in preventing dental caries^.111^. In addition to breastfeeding and bottle feeding, introducing comforting oral device, such as pacifier, has been found to serve as a potential risk factor for the colonization of *S. mutans* in infant’s mouth in early infancy.^112^

When considering the influence of feeding methods on infant oral microbiome, one limitation to be underlined is study groups from reported literatures were often not randomized, which might pose unknown group differences correlated to oral microbiota acquisition, in addition to the feeding methods. As a commonly limitation for non-randomized trials, these imbalanced factors between groups, e.g. race, ethnicity, age, gender, health conditions, caregivers’ oral and overall health, are confounding factors and could lead to biased results.

### Maternal influence

Colonization of oral mucosal surfaces begins at birth with the introduction of bacteria and fungi through a variety of paths, including maternal perineum–infant oral transmission during childbirth,^23^ parental exposures, digit sucking, diet, and horizontal transmission from caregivers and peers.^29–31^ Strain-resolved metagenomic profiling confirms transient maternal transmission of strains originating from multiple sources, with the vaginal, skin, oral, and gut communities all contributing to the early infant microbiome.^113^ However, a few days after birth, the contribution from the vaginal and skin microbiome have decreased, while gut microbes from the mom persist in the infant’s gut presumably through oral entry.^113^ By day 3 of life, 95% of the infant’s oral microbiome was shared with maternal microbiomes.^113^ Although evidence supports the maternal–neonate transmission of pathogenic microorganisms, e.g., *Candida* species immediately after birth into early infancy,^114,115^ more importantly, the maternal–neonatal transmission of microbes also serves an evolutionary survival function by preparing the infant to adapt to the environment by prepping the sterile infant gut with maternal microbes.^116,117^ Acquisition of maternal microbes gives the infant gut a head start through colonization with favorable symbiotic microbes, potentially protecting the infant from life-threatening respiratory and diarrheal infections that were a major cause of infant mortality in the early twentieth century and remain an important cause in some developing countries.

As a significant number of the human-body early colonizer are of maternal origin, we have recently compared the oral microbiota in a set of mother and preschool children, in which the children were grouped, based on their caries status, into severe early childhood caries (ECC) or caries-free. We observed that the microbial community structure and diversity within the mother–child dyads were similar (although species abundance differed across pairs) despite the caries status. Our observed strong maternal influence on the oral microbiota of children is supported by the recent observations of Chu et al.^118^, who found that the infant and maternal oral microbiota shared a similar community structure and taxonomic membership. Another study by Mason et al. revealed that 85% of infants’ oral microbiota resemble their mothers’ in the first six months after birth, suggesting maternal oral microbiota plays a role in seeding microbial species to the child. However, intriguingly, the mother–child oral microbiome similarity significantly decreased with the eruption of primary teeth in a child’s mouth, often, around 6–8 months.^29^

Similar to the bacterial community, maternal prints have been found in the infant oral mycobiomes.^32^ Infant and maternal oral mycobiomes were similar to each other when alpha and beta diversities were compared; the total numbers of observed taxa were comparable in infant and adult samples for throughout longitudinal observational points, regardless of body site (oral, skin, and anal).^32^ Among fungal species present in the oral cavity, *C. albicans* is the most prevalent species. *C. albicans* colonizes in the oral cavity of neonates as well, both vertical and horizontal transmission was implied to their emergence. The vertical transmission ranges from 14% to 41% when measured by different methods, e.g., electrophoretic karyotyping, restriction endonuclease analysis of genomic DNA, and DNA fingerprinting.^119,120^ Our study examined the relatedness of *C. albicans* among 18 preschool children and mother dyads using restriction endonuclease analysis of the *C*. *albicans* genome and found more than 60% of the child–mother dyads harbored identical *C*. *albicans* strains.^121^ While no caspofungin-resistant strains were identified when compared to the wild type, *C*. *albicans* isolated from more than 65% of child–mother dyads demonstrated similar susceptibility to the antifungal medication caspofungin.^121^

## Health implications in children’s oral microbiome

The oral microbiome remains its stability over time in healthy individuals, despite subjected to a variety of host and environmental challenges. The distinct oral microbial community is associated with a series of oral and systematic diseases.^10,122–128^ Although a majority of these studies are cross-sectional or case–control designed, with small sample sets which are incapable of establishing causative relationship between oral microbiome and diseases, changes in the characteristics of the oral microbiota may provide correlative insight and projection into the onset, progression, and recurrence of human diseases. Here, we further zoomed into the children’s health and summarized studies that have provided critical insights into the oral microbial markers of the oral and systemic health in children. The oral taxonomic characteristics associated with each disease status are summarized in Table 1.

**Table 1:** Oral microbiome implications and children’s diseases

Health condition in children	Oral microorganisms with increased relative abundance	Oral microorganisms with decrease relative abundance	Study type from which association identified
Early childhood caries	*Streptococcus mutans* *Streptococcus salivarius* *Streptococcus sobrinus* *Streptococcus parasanguinis* *Streptococcus wiggsiae* *Streptococcus exigua* *Lactobacillus salivarius* *Parascardovai denticolens Porphyromonas* *Veillonella* *Candida albicans*	*Actinomyces*	Case control^43^^,44^^,47,142^ Prospective cohort^42,46^
Celiac diseases	*Rothia* *Porphyromonas endodontalis Gemellaceae* *Prevotella nanceiensis* *Streptococcus sanguinis* *Lachnospiraceae*	*Actinobacteria* *Actinomyces* spp. *Atopobium* spp. *Corynebacterium durum*	Case control^39^
Autism	*Limnohabitans* spp. *Planctomycetales*	*Ramlibacter tataouinensis* *Mucilaginibacter* spp. *Bacteroides vulgatus Gemmata* spp.	Cross-sectional^149^
Henoch-schönlein purpura disease	*Neisseriales* *Neisseriaceae* *Neisseria* *Veillonella* *Nagativicutes* *Veillonellales* *Veillonellaceae* *Prevotella* *Prevotellaceae* *Bacteroidetes* *Bacteroidia* *Bacteroidales*	*Proteobacteria Gammaproteobacteria Pseudomonadales Moraxellaceae Acinetobacter Alphaproteobacteria Pasteurellaceae Pasteurellales* *Haemophilus*	Case control^151^
Pediatric appendicitis	*Pasteurella stomatis*	*Eikenella corrodens* *Fusobacterium nucleatum*	Cross-sectional^49^
Pediatric inflammatory bowel disease	*Spirochaetes* *Synergistetes* *Bacteroidetes*	*Fusobacteria* *Firmicutes*	Case control^50^
Pediatric obstructive sleep apnea syndrome	*Veillonella* *Prevotella* *Mogibacterium* *Campylobacter* *Butyrivibrio*	*Thermus* *Pseudomonas* *Lautropia* *Achromobacter*	Case control^164^

### Oral microbiome and early childhood caries (ECC)

Although largely preventable, ECC remains the single most common chronic childhood disease, with nearly 1.8 billion new cases per year globally.^129,130^ The microbial etiology of ECC is linked with poly-bacterial infection of teeth. Traditionally, *S. mutans* is considered as a prime culprit for ECC due to its acidogenicity, aciduricity, and ability to form extracellular glucans.^131–137^ Although at very low levels, *S. mutans* was detected in the oral cavity of the infants in early infancy, even before tooth eruption;^138^ and with a trend of the increasing amount with the presence of teeth and notably higher in children with ECC.^59^ Interestingly, a recent analysis of calcified dental plaque shows that the composition of oral microbiota remained relatively constant between Neolithic and Medieval eras. Cariogenic bacteria (e.g., *S. mutans*) became dominant beginning with a major production of processed flour and sugar, beginning with the industrial revolution.^139^ Well-designed longitudinal studies have demonstrated the predictive power of using *S. mutans* to predict ECC risk. Fontana et al.^140^ prospectively followed a cohort of 329 US children ((26 ± 6) months old) for 1 year and reported that children with more than 10^5^ colony-forming unit per mL salivary *S. mutans* at the baseline were at higher risk for developing ECC. Piva et al.^141^ evaluated a cohort of 163 Brazilian children (3–4 years old) in a 2-year prospective period, and found higher *S. mutans* counts were associated with caries progression.

In addition to *S. mutans*, several studies have characterized the oral microbiota in caries-active children, and have identified additional species that are associated with caries state, including *S. salivarius, S. sobrinus*, *S. parasanguinis, S. wiggsiae*, *S. exigua, L. salivarius, Parascardovai denticolens, Porphyromonas, Actinomyces,* and *Veillonella.*^42–47^ Gross et al.^42^ sequenced the plaque samples from 36 severe ECC (S-ECC) and 36 caries-free (CF) children with a mean age of 23.6 months and monitored the microbiota evolve during the onset of S-ECC. They confirmed that *S. mutans* was the dominant species in many, but not all children with S-ECC.^42^ Other species that had elevated quantities among children with S-ECC were *S. salivarius, S. sobrinus*, and *S. parasanguinis*. Elevated levels of these species were observed specifically among children with no or low levels of *S. mutans*, suggesting these species were playing alternative pathogenic roles, and that targeting species in addition to *S. mutans* may be promising interventions for ECC and S-ECC.^42^ Among children without past caries history, *Veillonella*, not *S. mutans* or other acid-producing species, were found to be a predictor for future caries.^42^ The levels of *Veillonella* highly correlated with total acid-producing species.^42^ The underline explanation of the phenome lies in that *Veillonella* is well-known for metabolizing lactate; lactate, in turn, is an end-product from the carbohydrates-derived catabolism by *Streptococcus* species that many of them are associated with caries. An inspiring way to elaborate is that *Veillonella* might not be acting as a criminal for causing caries, but a “whistleblower” for caries. On the contrary, with the occurrence of caries and advancement of caries stages, the abundance of specific taxa reduced, for instance, *Streptococcus mitis* group, *Neisseria*, and *S. sanguinis.*^42^ In concert with the different abundance of cariogenic and symbiotic bacteria in caries and healthy children, community diversity was also reduced in children with caries as compared to their healthy counterparts.^42^

Intriguingly, available microbiological data also show that this pediatric oral disease, ECC/S-ECC, has a strong fungal involvement. Particularly, *Candida* species (especially *C. albicans*) are more prevalent in the oral cavity of children with ECC or S-ECC (over 80%) in comparison to caries free children (approximately 20%), and a higher abundance in the ECC/S-ECC group; the carriage of the fungus is positively correlated with the carriage of *S. mutans* and the severity of dental caries in children, as reviewed by Xiao et al.^142^. The presence of *C. albicans* in preschool children’s oral cavity is associated with a 6.7 times higher risk of experiencing ECC than those ones do not have this fungal species.^142^ Our recent findings^48^ demonstrated that the presence of *C. albicans* is further associated with alterations of oral bacterial composition and cariogenic bacteria virulence that could contribute to an oral environment that are more prone to caries. The *C. albicans*-associated oral bacteriome is characterized as an enrichment of a highly acidogenic–aciduric bacterial community, with an increased abundance of plaque *Streptococcus* (particularly *S. mutans*), certain species of *Lactobacillus/Scardovia* and salivary/plaque *Veillonella* and *Prevotella*, and decreased levels of salivary/plaque *Actinomyces.*^48^ Following the enrichment of *S. mutans* in plaque, the enzymatic activity of glucosyltransferases, key enzymes that could synthesize extracellular matrix by *S. mutans*, was also enhanced. Our finding from the clinical setting was verified in rodent models by Bowen and Koo,^143^ indicating that *C. albicans* could play an essential role in enhancing plaque glucosyltransferase enzymatic activities and caries pathogenesis. Therefore, in addition to cariogenic bacteriome, the need for including fungal counterparts in oral microbiome research in the context of ECC/S-ECC, and understanding its role in the onset, progression, and relapse of this disease in future longitudinal studies are paramount, by doing so could potentially lead the way to prevent and treat this costly and intractable disease from an innovative fungal perspective.

Besides cariogenic bacteria and yeast, *S. sanguinis* has received much attention in the context of caries prevention. *S. sanguinis* starts its oral colonization in the second half of the first year of infants, in association to tooth emergence,^144^ following a similar pattern of development regardless of children’s caries experience.^59^ Researchers believe *S. sanguinis* could be playing a benign role in the oral cavity, and have an antagonistic role in dental caries since it may compete with cariogenic *S. mutans* for colonization sites on tooth surface.^144^

### Association between children’s oral microbiome and systematic diseases

#### Infant oral microbiome and weight gain

The oral microbiome may play a vital role and serve as an indicator of infant development. A recent study^40^ revealed that oral microbiome harbors a better bacterial signature for predicting the weight gain in the early infancy when compared to the gut microbiome. In this study, via 16S rRNA sequencing, the authors analyzed the gut and oral microbiota of 226 young children at seven times points in the first two years of life, along with data collection on infant rapid weight gain.^40^ Specifically, parameters on infant’s weight and body length were collected to identify infants with rapid weight gain, and to derive growth curves with innovative Functional Data Analysis techniques.^40^ Authors revealed that growth curves were negatively associated with the oral microbial diversity, and positively associated with the *Firmicutes*-to-*Bacteroidetes* ratio of the oral microbiota.^40^ The study results suggest for the first time that the association between the oral microbiota and the temporal pattern of weight gain in early childhood might be stronger and more consequential than previously thought, and thus requires further characterization.^40^ However, the mechanism underlying these associations remains unknown.

#### Oral microbiome and celiac disease (CD) in children

CD, a chronic immune-mediated enteropathy, affects the small intestinal mucosa after the ingestion of gluten from wheat, rye, and their cross-related varieties in genetically susceptible individuals.^145^ The estimated prevalence of pediatric CD is between 1/100 and 1/400 (ref. 146). The symptoms of CD in children are characterized by the occurrence of diarrhea, failure to thrive, and abdominal bloating in young infants in the months following gluten introduction.^146^ Francavilla et al. examined the salivary microbiome and metabolome of 13 CD children with gluten-free diets (T-CD) and their healthy counterparties (HC). Their results suggest that CD is associated with dysbiosis of oral microbiota that could lead to oral metabolome. The salivary level of total cultivable anaerobes significantly (*P* < 0.05) differed between the T-CD and HC children. Children with T-CD had less diverse salivary microbiome and increased abundance of *Rothia*, *Porphyromonas endodontalis, Gemellaceae, Prevotella nanceiensis, S. sanguinis*, and *Lachnospiraceae* compared to their healthy controls.^147^ Children with T-CD had a decreased abundance of *Actinobacteria, Actinomyces* spp.*, Atopobium* spp., and *Corynebacterium durum*. The relative abundances of these taxa are consistent with higher levels of organic volatile compounds (e.g., ethyl-acetate, nonanal, and 2-hexanone) in the saliva of affected children.^147^

#### Oral microbiome and autism in children

Autism spectrum disorder (ASD) reflects a different level of symptom severity in two core domains: deficits in social communication and interaction, and restricted repetitive behaviors.^148^ ASD occurs in all racial, ethnic, and socioeconomic groups and is more prevalent among boys.^148^ According to the Center for Disease Control and the Autism and Developmental Disabilities Monitoring Network in the USA, the prevalence is 1 in 88 children.^148^ With the ASD etiology remaining puzzled, it is particularly important to diagnose and provide appropriate interventions to diseased children at an earlier time point. ASD is associated with several oropharyngeal abnormalities, including buccal sensory sensitivity, taste and texture aversions, speech apraxia, and salivary transcriptome alterations. Alterations in the gut microbiome have been established as features of ASD, which was speculated to impact individual’s behavior via the “microbial–gut–brain axis”.

To uncover the oral microbiome alterations in children with ASD, Hicks et al.^149^ used shotgun meta-transcriptomic data to identify changes in the salivary microbiome of 348 preschool children who were 2–6 years of age. These children were grouped into three developmental profiles: children with ASD, children with nonautistic developmental delay (DD), and children with typically developing (TD). This research group found five distinguished microbial ratios between ASD and TD children (79.5% accuracy), three distinguished microbial ratios between ASD and DD (76.5% accuracy), and three distinguished microbial ratios between ASD children with and without gastrointestinal disturbance (85.7% accuracy). When comparing the taxa between ASD and TD children, the abundance of two taxa were elevated in ASD children, they were *Limnohabitans* sp. 63ED37-2 with a false discovery rate (FDR) at 0.01 and *Planctomycetales*, with a FDR at 0.04. The abundance of four taxa were decreased in ASD children, particulary, *Ramlibacter tataouinensis* TTB310 with an FDR at 0.001, *Mucilaginibacter* sp. PAMC 26640 with an FDR at 0.001, *Bacteroides vulgatus* with an FDR at 0.05, and *Gemmata* sp. SH-PL17 with an FDR at 0.05. Furthermore, when the taxa abudance between ASD and DD children were compared, two taxa were elevated in children with ASD, *Brucella* (FDR = 0.05) and *Enterococcus faecalis* OG1RF (FDR = 0.05), while one taxa (*Flavobacterium* sp. PK15, FDR = 0.05) was decreased in ASD children. The study results indicate that gut microbiome disruption in ASD could extend to the oropharynx. Future routine assessment of children’s oral microbiome could be developed as a non-invasive and possible sensitive tool to diagnose ASD and assess its progression status.

#### Oral microbiome and Henoch-Schönlein purpura disease in children

As the most common form of vasculitis in children, Henoch-Schönlein Purpura (HSP) causes inflammation and bleeding in the small blood vessels of the skin, joints, intestines, and kidney.^150^ Over 75% of children diagnosed with HSP were younger than 10 years old, with a peak incidence at 4–6 years of age.^150^

In a case–control study, Chen et al.^151^ examined the association between oral microbiota and HSP by sequencing the 16S rRNA genes of oral samples from 98 HSP children and their 66 healthy counterparts. Distinctive patterns of oral microbiota were seen between the healthy children and those with HSP, higher oral microbial diversity and richness were observed in Children with HSP compared to their controls. Using a linear discriminant analysis (LDA) effect size (LEfSe) algorithm, Chen et al.^151^ further detected 21 bacterial taxonomic clades showing statistical differences in children with HSP, with 12 increased and 9 decreased taxa. The 12 genera with increased abundance were *Neisseriales, Neisseriaceae, Neisseria, Veillonella, Nagativicutes, Veillonellales, Veillonellaceae, Prevotella, Prevotellaceae, Bacteroidetes, Bacteroidia*, and *Bacteroidales*. The nine genera with decreased abundance were *Proteobacteria, Gammaproteobacteria, Pseudomonadales, Moraxellaceae, Acinetobacter, Alphaproteobacteria, Pasteurellaceae, Pasteurellales*, and *Haemophilus*.

Notably, the relative abundance of several taxa correlated with clinical manifestations of HSP. The more prolonged hospital stay was associated with a lower abundance of *Butyrivibrio* sp, but a higher abundance of *Haemophilus* sp.^151^ *Haemophilus* sp was also negatively correlated to the amount of IgE and IgM.^151^ *Prevotella* positively correlated with the amount of IgM.^151^ *Prevotella nanceiensis* was more abundant in children with HSP and positively correlated with the amount of IgA.^151^ These study findings that children with HSP have significantly different oral microbiota and a particular association between taxa abundance and HSP clinical parameters exist, suggest the potential of using the oral microbial signatures to identify high-risk populations for HSP and predict clinical progress of HSP children. Although this study does not imply causality between oral microbiota changes and HSP, it provides insight into identifying the types and pathways of bacteria that can be used to predict, prevent, or treat HSP.

#### Oral microbiome and pediatric appendicitis

Appendicitis is the most common disease that demands urgent surgery among pediatric patients.^152^ The lifetime risk of developing appendicitis is 7% in girls and 9% in boys.^152^ Symptoms of appendicitis could range from abdominal pain and vomiting caused by mild or moderate inflammation to life-threatening conditions caused by appendix perforation with extensive contamination.^152^ Pediatric perforated appendicitis rates are approximately 30% (20%–74%).^153^ Since younger children have difficulty to articulate their symptoms, a much higher perforation rate is seen among younger children. A cross-sectional study data revealed that the appendix preformation occurred among 100% patients less than 1-year-old and 69% patients at 5 years of age.^154^

To understand the potential role of oral microbial in pediatric appendicitis, Blod et al.^49^ rofiled the microbes from inflamed appendices and the gingival sulcus of 21 children who were undergoing appendectomy for acute appendicitis and 28 healthy controls using 16S rRNA sequencing. In addition to profiling the microbial community using sequencing, quantitative measures using RT-qPCR was performed to assess the presence of *Fusobacterium nucleatum*, *Peptostreptococcus stomatis*, and *Eikenella corrodens*. Furthermore, authors used an acid-killing assay to examine these bacteria’s viability to mimic gastric challenge. Although *P. stomatis*, *E. corrodens*, and *F. nucleatum* were detected in both appendicitis and healthy children, more viable *P. stomatis* were obbserved in the gingival sulci of children with acute appendicitis compared the healthy controls. For children with acute appendicitis, less viable *E. corrodens* and *F. nucleatum* presented in inflamed appendices than those found in the gingival sulci. As the oral cavity is the entry port of the gastrointestinal system, the authors proposed a possible oral-gastrointestinal migration of oral bacteria, and subsequently suggest the oral cavity could be a relevant microbial reservoir for developing acute appendicitis.

#### Oral microbiome and pediatric inflammatory bowel disease

Inflammatory bowel disease (IBD) is a chronic inflammatory disease of the gastrointestinal tract, likely caused by an aberrant immune response to the microbiota and other environmental challenges in genetically susceptible individuals.^155^ Oral mucosal inflammation is commonly noted in patients with IBD, particularly Crohn’s disease (CD). In all, 0.5%–80% of adult patients with CD manifest oral pathology.^156,157^ In children, 42% of new diagnoses of CD had oral manifestations.^158^ Docktor et al.^50^ examined the oral microbiome (swab samples taken from the tongue and buccal mucosa) from a total of 114 children with CD, ulcerative colitis (UC), and healthy controls, and showed an overall decreased alpha diversity of oral bacterial community in children with CD when compared with healthy controls. In contrast, overall diversity of children with UC did not differ from the healthy controls.

When comparing the tongue samples collected from the CD children to healthy children, several key phyla were significantly reduced, *Fusobacteria* and *Firmicutes.*^50^ When comparing the tongue samples taken from the UC children to the control group, researchers noted a decrease in *Fusobacteria* in UC children, but an enrichment in *Spirochaetes, Synergistetes*, and *Bacteroidetes.*^50^ No individual phyla from the buccal mucosa samples were significantly different between CD/UC patients and their healthy controls.^50^ The loss of *Fusobacteria* and *Firmicutes* in children with CD were resonated in studies examining the intestinal microbiome.^156,158,159^ Docktor et al.^50^ commented that with the prevalence of oral pathology in CD and the ease of oral mucosal sampling, further study could explore the potential of using the oral microbiome as a diagnostic and prognostic tool for pediatric IBD.

#### Oral microbiome and pediatric obstructive sleep apnea syndrome

As a breathing disorder during sleep, obstructive sleep apnea syndrome (OSAS) is characterized by recurrent episodes of complete or partial upper airway obstruction that interrupt nocturnal ventilation and alter normal sleep patterns.^160–162^ OSAS occurs among children of all age, including infants, with a peak incidence between 3 and 6 years of age.^161^ The prevalence of pediatric OSAS has been estimated to be approximately 3%.^163^ While associations between oral diseases and OSAS in the adult population were observed in several cross-sectional studies, limited research reported potential association among the pediatric population.

In a case–control study, Xu et al.^164^ compared the oral microbiome composition between 30 children OSAS and 30 health counterparts using 16S rRNA gene sequencing. Their results indicate that the oral microbiome composition was significantly different in pediatric OSAS compared to their healthy controls. *Thermus, Pseudomonas, Lautropia*, and *Achromobacter* showed higher abundances in the normal control group, whereas *Veillonella, Prevotella, Mogibacterium, Campylobacter*, and *Butyrivibrio* were more abundant in the patients with OSAS. In complementary to comparing the oral microbiome, this study group analyzed the urinary metabolome of the study participants and revealed that specific oral microbial changes were correlated with urinary metabolite perturbations in pediatric OSAS. However, the further causal relationship needs examination in longitudinally designed cohort studies. An additional concern lies that there is now ample data confirming that OSAS associated with obesity is highly prevalent in children.^165^ Whether the imbalanced oral microbiome demonstrated between children with and without OSAS is due to obesity or imbalanced diet that led to obesity remains unknown.

## Limitations of current evidence and summary

The mouth is the portal to the gut. Its microbial ecology represents a possible marker if not a risk factor for the disease. The recent advances in salivary biomarker diagnostics and oral microbiome analyses have broadened the discovery of microbial pathogens associated with oral and systematic diseases (e.g., dental caries, periodontal disease, autoimmune diseases, diabetes, and cancers). Although more attention has been paid to the association between gut microbiota and overall health, the oral microbiota has shown its relevance and possibility of being surrogates of gut microbiota, which could provide equivalent dialogistic power with better handling. Compared to the gut microbiome sampling, oral sampling is more psychologically acceptable more easily accessed by patients, as well as by the healthcare professional. Among vulnerable populations, such as infants and young children, oral samples make the perfect diagnostic medium due to its noninvasive and easy handling properties, which holds great promise to be used as diagnostic tools.

With the above-mentioned disease-diagnostic promises, major limitations of establishing the associations between the oral microbiome and children’s diseases lie in:

1. The currently available evidence from the majority of the oral microbiome studies are cross-sectional or case–control designed with a small sample size, which makes it impossible to establish causative associations between oral microbial community and diseases. For example, with the exception of the well-established association established between *S. mutans* and ECC from various of existing studies across case–control and prospective studies,^42–47^ studies that examined the oral microbiome of children with systemic diseases including pediatric autism, irritable bowel syndrome, pediatric appendicitis, CDs, etc., are lack of power to imply causal relationships between the oral microbiome and the diseases due to the limitation of the study design. Thus, it is not clear whether the change in oral microbial community is a predictor of future disease, or a result of systemic diseases.
2. The current childhood microbiome studies based exclusively on 16S rRNA amplicon sequencing and are therefore limited to the assessment of taxonomic composition and diversity of the microbiome. A detailed metagenomic analysis of microbiota functions that contribute to host-microbiome interactions within the oral environment is needed to identify the mechanistic basis of these interactions and identify metabolic or virulence pathways as therapeutic targets.
3. The majority of oral microbiome studies are focused solely on the bacterial community and do not address critical contributions of the fungal and viral members of the oral microbial community to oral health and disease. Since cross-kingdom interactions have received applause in recent years, given multiple cross-kingdom interactions have been identified (bacteriome and fungi, host and fungi, for say), future research should consider examining the interaction between different microbial community and their interaction with the host.
4. Oral microbiome studies among healthy adults indicate that the diversity of the oral microbiome varies by geography and race/ethnicity^166^ including inter-social group variation.^167^ However, few studies have addressed the impact of racial diversity on oral microbiome development in early infancy.
5. Lack of standardized procedure for oral sample collection. To enable microbiota data comparison, standardization of each step in the process for sequencing data generation and analysis is critical. These procedures span from clinical sampling, sample handling and storing, sequencing, bioinformatic data processing, to taxonomic interpretation. One of the most critical factors of the upstream of the process is the oral sample collection. The timing of the sampling (morning, afternoon, before, or after children’s feeding), sample collection tools, sample storing are all critical factors that can induce a significant difference in downstream results. Our collective experience from birth cohort studies that require oral sampling from newborns to toddlers suggests sampling in young children, particularly in toddlers, is even more challenging due to their mobility and limited cooperation. Non-standardized sampling methods could lead to an inherently biased study and challenge the data comparability across study time points internally and across external studies. The development of standardized sample collection methods is urgently needed and would facilitate a qualified comparison of data in the field.

In summary, the complex interplay between the oral microbiome and microbiomes colonized at other body parts in early infancy, host immune factors, and health, suggests complex bidirectional, non-linear interactions that make causality challenging to tease apart making this a very fruitful area of scientific inquiry. The future use of oral microbiome to advance human health will depend on further validation of disease-specific microbial biomarkers and their incorporation into diagnostic and preventive programs that are sensitive and specific, rapid in result delivery and cost-effective for broad implementation. With the complementary of human genomics, proteomics, transcriptomics, metabolomics, the children’s oral microbiome may stand in the center stage of the future precision medicine and personalized medicine.
